# Supplementary material for: Solution-Processable Colorless Polyimides Derived from Hydrogenated Pyromellitic Dianhydride: Strategies to Reduce the Coefficients of Thermal Expansion by Maximizing the Spontaneous Chain Orientation Behavior during Solution Casting
Source: Polymers (Basel). 2022 Mar 11;14(6):1131. doi: 10.3390/polym14061131 (PMC8950016; doi:10.3390/polym14061131)
Supplement: Supplementary file 1 [file polymers-14-01131-s001.zip › polymers-1599843-supplementary-done.pdf]

polymers MDPI

Supporting Information

S1. Synthetic procedures for a monoamide-type diamine (PDA-*t*CHDCA).

In a sealed flask, *trans*-1,4-cyclohexanedicabonyl chloride (*t*-CHDCC, 10 mmol) was dissolved in anhydrous tetrahydrofuran (THF, 5.4 mL). In another sealed flask, 4-nitroaniline (4-NA, 23 mmol) was dissolved in THF (8.2 mL) in the presence of pyridine (30 mmol, 2.4 mL) as an HCl acceptor. To the *t*-CHDCC solution cooled at 0 °C, the 4-NA solution was gradually added using a syringe with continuous magnetic stirring, after which the reaction mixture was stirred at 0 °C for several hours, subsequently at room temperature for 12 h. The pale-yellowish precipitate formed was collected by filtration, washed with THF, a large quantity of water, and methanol, and dried at 120 °C under vacuum for 12 h (yield: 93%). The crude product was recrystallized from *N*,*N*-dimethylformamide (DMF), washed with ethanol, and dried at 100 °C for 12 h under vacuum (recrystallization yield: 81%). The product was confirmed to be the desired dinitro compound by the following data. FT-IR (KBr plate method, cm^{-1}): 3320/3287/3221 (amide N–H), 3090 (C_{arom}–H), 2945/2867 (C_{aliph}–H), 1677 (amide-I, C=O), 1555 (NO_2 + amide-II, C=O), 1498 (1,4-phenylene), 1337 (NO_2). ^{1}H-NMR [400 MHz, dimethyl sulfoxide (DMSO)-d_6, δ, ppm]: 10.53 [s, 2H (relative integrated intensity: 2.00H), NHCO], 8.22 [d, 4H (4.04H), J = 8.6 Hz, 2,2′,6,6′-protons of the terminal nitrobenzene units (NB)], 7.86 [d, 4H (4.03H), J = 8.7 Hz, 3,3′5,5′-protons of NB], 2.43 [t (unresolved), 2H (2.02H), 1,4-protons of the central cyclohexane unit (CHx)], 1.98 [q, 4H (3.99H), J = 7.4 Hz, 2,3,5,6-axial protons of CHx], 1.53–1.48 [m, 4H (4.04H), 2,3,5,6-equatorial protons of CHx],

The catalytic reduction of this dinitro compound was conducted while tracking the reaction with thin-layer chromatography as follows. The nitro compound (3.09 g) was dissolved in DMF (300 mL) in the presence of Pd/C (0.38 g) as a catalyst. The reaction mixture was refluxed at 80 °C for 5 h in a hydrogen atmosphere, and the catalyst residue was removed by hot filtration. The filtrate was concentrated with an evaporator and slowly poured into a large quantity of water. The white precipitate formed was washed with methanol, and dried at 120 °C for 12 h under vacuum (yield: 52%). The product was confirmed to be the desired diamine (PDA-*t*CHDCA) by the following data. Melting point (DSC): 385 °C. FT-IR (KBr plate method, cm^{-1}): 3376/3294/3222/3172 (amine + amide N–H), 3034 (C_{arom}–H), 2952/2915/2853 (C_{aliph}–H), 1670/1536 (amide C=O), 1509 (1,4-phenylene). ^{1}H-NMR (400 MHz, DMSO-d_6, δ, ppm): 9.41 [s, 2H (2.00H), NHCO], 7.22 [d, 2H (4.00H), J = 8.8 Hz, 3,3′,5,5′-protons of the terminal aniline unit (AN)], 6.47 [d, 4H (4.01H), J = 8.8 Hz, 2,2′,6,6′-protons of AN], 4.81 [s, 4H (4.07H), NH_2], 2.25 [t (unresolved), 2H (2.02H), 1,4-protons of CHx], 1.85 [q, 4H (4.05H), J = 7.0 Hz, 2,3,5,6-axial protons of CHx], 1.47–1.42 [m, 4H (3.94H), 2,3,5,6-equatorial protons of CHx]. Elemental analysis, Anal. Calcd (%) for $C_{20}H_{24}O_2N_4$ (352.44): C, 68.16; H, 6.86; N, 15.90. Found: C, 67.99; H, 6.65; N, 16.03.

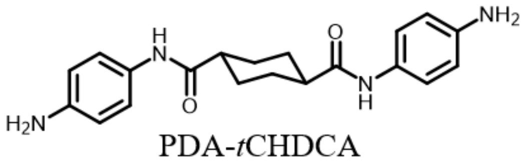

PDA-*t*CHDCA

Table S1. Abbreviations, commercial sources, and melting points of the common monomers used in this study.

Common diamines	Source	Vacuum-drying condition	Melting point [a] (°C)
4,4′-Oxydianiline (4,4′-ODA)	Wako Chemical	50 °C/24 h	191 [a]
3,4′-Oxydianiline (3,4′-ODA)	Wakayama Seika	50 °C/12 h	79 [a]
p-Phenylenediamine (*p*-PDA)	Wako Chemical	30 °C/24 h	141 [a]
m-Tolidine (*m*-TOL)	Wako Chemical	50 °C/24 h	106 [b]
4,4′-Diaminobenzanilide (DABA)	Tokyo Chemical Industry (TCI)	50 °C/12 h	205 [a]
4-Aminophenyl-4′-aminobenzoate (APAB)	Wakayama Seika	50 °C/12 h	185 [a]
4-Amino-2-methylphenyl-4′-aminobenzoate (ATAB)	Wakayama Seika	50 °C/12 h	162 [a]
2,2′-Bis(trifluoromethyl)benzidine (TFMB)	Wakayama Seika	50 °C/12 h	184 [a]
2,2-Bis[4-(4-aminophenoxy)phenyl]propane (BAPP)	Wakayama Seika	50 °C/12 h	130 [a]
1-*exo*,2*exo*,4*exo*,5*exo*-Cyclohexanetetracarboxylic dianhydride (1*S*,2*R*,4*S*,5*R*-Cyclohexanetetracarboxylic dianhydride) (H-PMDA)	Mitsubishi Gas Chemical	160 °C/12 h	303 [a]

[a] Data determined from the endothermic peak measured at a heating rate of 5 °C min^{-1} on DSC.
[b] Data from the safety data sheet

Table S2. Abbreviations, commercial sources, and melting points of the raw materials used in this study.

Raw materials	Source	Melting point (°C)
4-Nitrobenzoyl chloride (4-NBC)	TCI	73 [b]
2-Methoxy-4-nitroaniline	TCI	140 [b]
2-Methyl-4-nitroaniline	TCI	132 [b]
4-Nitroaniline	TCI	148 [b]
3-Methyl-4-nitrobenzoic acid	TCI	217 [b]
trans-1,4-Cyclohexanedicarbonyl chloride (*t*-CHDCC)	Iharanikkei Chemical Industry	
1-*exo*,2-*exo*,4-*exo*-Cyclohexanetricarboxylic anhydride (HTA)	Mitsubishi Gas Chemical	156 [a]
4-Aminobenzoic acid	TCI	189 [b]

[a] Data determined from the endothermic peak measured at a heating rate of 5 °C min^{-1} on DSC.
[b] Data from the safety data sheet

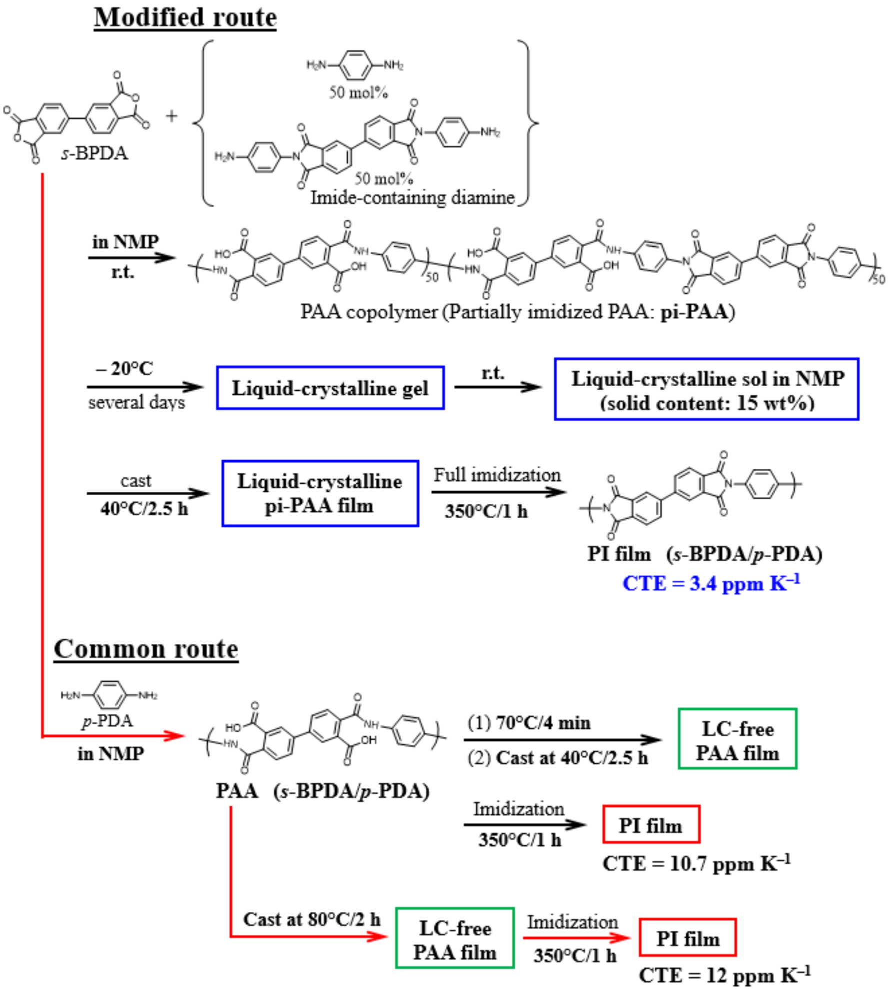

Figure S1. Different film preparation routes via full thermal imidization of the partially imidized *s*-BPDA/*p*-PDA cast film with liquid-crystalline (LC) domains and the pristine PAA cast film without LC domains.
